# Associations among Screen Time and Unhealthy Behaviors, Academic Performance, and Well-Being in Chinese Adolescents

**DOI:** 10.3390/ijerph14060596

**Published:** 2017-06-04

**Authors:** Hanyi Yan, Rui Zhang, Theresa M. Oniffrey, Guoxun Chen, Yueqiao Wang, Yingru Wu, Xinge Zhang, Quan Wang, Lu Ma, Rui Li, Justin B. Moore

**Affiliations:** 1 School of Health Sciences, Wuhan University, Wuhan 430071, China; yanlq7@hotmail.com (H.Y.); elle529@126.com (Y.W.); yingru.wu@hotmail.com (Y.W.); ylcz2920@126.com (X.Z.); wangquan73@whu.edu.cn (Q.W.); malu@whu.edu.cn (L.M.); 2 College of Life Sciences, South-Central University for Nationalities, Wuhan 430074, China; zhangrui@mail.scuec.edu.cn; 3 Cerus Consulting LLC, Winston-Salem, NC 27101, USA; toniffrey@gmail.com; 4 Department of Nutrition, The University of Tennessee at Knoxville, Knoxville, TN 37996, USA; gchen6@utk.edu; 5 Wake Forest Baptist Medical Center, Department of Family & Community Medicine, Wake Forest School of Medicine, Medical Center Boulevard, Winston-Salem, NC 27157, USA; jusmoore@wakehealth.edu; 6 Department of Epidemiology & Prevention, Wake Forest School of Medicine, Medical Center Boulevard, Winston-Salem, NC 27157, USA

**Keywords:** screen time, unhealthy eating behaviors, academic performance, mental health, Chinese adolescents

## Abstract

Screen time is negatively associated with markers of health in western youth, but very little is known about these relationships in Chinese youth. Middle-school and high-school students (*n* = 2625) in Wuhan, China, completed questionnaires assessing demographics, health behaviors, and self-perceptions in spring/summer 2016. Linear and logistic regression analyses were conducted to determine whether, after adjustment for covariates, screen time was associated with body mass index (BMI), eating behaviors, average nightly hours of sleep, physical activity (PA), academic performance, and psychological states. Watching television on school days was negatively associated with academic performance, PA, anxiety, and life satisfaction. Television viewing on non-school days was positively associated with sleep duration. Playing electronic games was positively associated with snacking at night and less frequently eating breakfast, and negatively associated with sleep duration and self-esteem. Receiving electronic news and study materials on non-school days was negatively associated with PA, but on school days, was positively associated with anxiety. Using social networking sites was negatively associated with academic performance, but positively associated with BMI z-score, PA and anxiety. Screen time in adolescents is associated with unhealthy behaviors and undesirable psychological states that can contribute to poor quality of life.

## 1. Introduction

Healthy behaviors are learned in adolescence [1]. Healthy eating, physical activity (PA), and positive self-perceptions are important for quality of life and physical health [2]. Screen-based behaviors (e.g., television viewing and using social networking sites) are key leisure activities among adolescents [3], and the cumulative time spent engaged in these behaviors is often thought of as a youth risk behavior [4,5,6]. Population estimates show that between 40% and 80% of young people exceed screen time recommendations [7,8].

Since the establishment of recommendations for pediatric screen times [3], an increase in the number and volume of sedentary activities negatively impacting positive behaviors [9] and overall wellbeing [10] has been observed. These effects include less time for PA and sleep [9], poorer academic performance [11], and higher risk of obesity and becoming overweight [12]. Screen-based behaviors may have negative psychological effects [3], and may further impact health through behavioral mediators [3] such as unhealthy eating and PA habits [13,14].

Chinese youth experience many cultural influences that may promote screen-based behaviors more than for their western peers. For example, many adolescents in China and other eastern Asian countries attend “cram schools” (i.e., test preparation centers) or employ private teachers to enhance their academic achievement [15]. The additional time spent in class, tutoring, or studying may limit the available time for PA or sleep and promote screen-based behaviors such as information seeking on the internet, which may negatively affect academic performance [15]. A study conducted in Beijing, China, showed that using computers, watching TV and playing e-games for more than two hours per day influenced middle school students to become overweight [16]. Other studies indicated that, for a number of reasons, Chinese teenagers did not spend enough time on PA [17,18,19], which may have led to obesity or certain mental diseases [18,20]. For Chinese adolescents aged from seven to 18, the prevalence of obesity increased from 1.63% in 1991 to 5.99% in 2011 (2.36% to 7.27% for boys and 1.40% to 4.64% for girls) [21].

Unfortunately, very few studies examining the impacts of screen-based behaviors have been conducted in Asian adolescents. Therefore, the aim of this study was to determine the amount of time adolescents in Wuhan, China, spent on screen-based behaviors, and the associations of this with adiposity, unhealthy eating behaviors, sleep, PA, academic performance, anxiety, self-esteem, and life satisfaction.

## 2. Materials and Methods

### 2.1. Study Population

A cross-sectional survey was conducted at two schools, a middle school and a high school, in Wuhan, Hubei, China, during the late spring/early summer of 2016. The study was conducted in accordance with the Declaration of Helsinki, and the protocol was approved by the Ethics Committee of Wuhan University (Project identification code 2016031269). All adolescents (*n* = 3059) enrolled in grades 7–12 were invited to participate in the study via a recruitment letter and consent form sent home to parents; written informed consent was obtained from a parent or guardian. For those providing consent, a survey was sent home to be completed and returned to school. Of those who consented, 149 respondents did not return the questionnaire, and 282 respondents did not provide essential information (i.e., grade, sex, eating behaviors, sleeping time and screen time variables). Two respondents older than 19 years of age and a respondent younger than 12 were excluded; this resulted in a final sample size of 2625 youths aged 13–18 years (86% of those contacted). All procedures were approved by the Wuhan University Ethics Board and the medical school district administrators.

### 2.2. Measures 

The school and grade of each student was recorded by study staff; date of birth, sex, height and weight were self-reported. Age, body mass index (BMI: weight in kg/height in m^2^), and sex and age standardized BMI (BMI z-score) were calculated [22].

Students were asked how many hours a day they usually spent watching television, playing e-games, receiving news or study materials from electronic devices, using social media sites or apps, and watching videos both on school days and on non-school days. Total hours per week of overall screen-based behaviors was calculated per type of screen-based behaviors. Response options referred to daily use (≤1, 2–3, 3–4, and >4 h/day). We collapsed “use, but not daily” and “do not use” [2] into four categories: “never”, “not every day”, “<1 h, 2–4 h”, and “>4 h daily”.

Academic performance was approximated by using an informal ranking scale based on students’ self-reports of scores on the last cumulative examination in their grade; options were top 20%, 20–40%, 40–60%, 60–80%, and lowest 20%. Answer selections were collapsed into two categories split at the median for analysis; the top 40% were coded as 1 and the last 60% were coded as 0.

Regarding unhealthy eating behaviors, students were asked how many times per week they skipped breakfast or had a late-night snack (after dinner). Answer options were none, 1, 2–3, 4–5, or all 7 days [23]. A dichotomous variable was created for skipping breakfast, to reflect consumption on all 7 days (coded as 1), as opposed to all other options (coded as 0) [24]. For late-night snacking, we created a dichotomous variable; participants who never snacked at night were coded as 1, and participants who reported snacking at night were coded as 0.

Regarding sleep duration, students were asked how many hours they typically slept at night on school days. The response options were <4, 5, 6, 7, 8, 9, or >10 h. A dichotomous variable was created with respondents who slept <8 h per night on school days being coded as 0, and those sleeping ≥8 h per night being coded as 1 [25].

Since strenuous PA is strongly [26] and independently associated with markers of cardio metabolic health [27], and can be more reliability assessed than light or moderate PA [28], we assessed only strenuous PA [28]. Students were asked whether they engaged in strenuous activity, equal to or more than three days a week (Yes = 1, No = 0). Strenuous activity was defined as sports, games, or dance that made them breathe hard, made their legs feel tired, or made them sweat [10].

The Middle School Student Mental Health Scale developed by Wang [29] was used to assess adolescents’ levels of anxiety. Participants were asked to quantify their anxiety during the previous seven days. The scale is comprised of six items scored on a five-point Likert-type scale. Responses were summed to derive an anxiety score ranging from 6 to 30 (with higher scores indicating higher levels of anxiety) [29].

The Satisfaction With Life Scale [30] was used to measure life satisfaction of participants. Students completed a seven-point Likert-type response scale in response to five items; answers were summed to create a life satisfaction score for each student. The possible range of scores was 5–35, with 20 representing the mid-point (5–19 indicating dissatisfied; 21–35 indicating satisfied) [31].

The Rosenberg Self-Esteem Scale [32] was used to assess participants’ self-esteem. Students answered 10 questions using a four-point Likert-type scale. Five items were positively worded and five were negatively worded. Items were summed with the negatively worded items reverse-coded to produce a self-esteem score from 0 to 30 (with higher scores indicating higher self-esteem) [33].

### 2.3. Statistical Analysis

All analyses were conducted using Stata 14 (StataCorp LLC, College Station, TX, USA). The distributions of each of the continuous outcome variables were assessed for normality. Because BMI was positively skewed, age and sex adjusted BMI z-scores [22] were calculated and dichotomized to overweight/obese (=0) or normal/underweight (=1). Because anxiety was positively skewed, a log transformation was applied.

Pearson’s chi-squared test (χ^2^) was conducted to examine differences in dependent variables by gender. Forced entry logistic regression analyses were used to examine the association between screen time (hours/week) and BMI z-score, unhealthy eating behaviors, sleep, PA, and academic performance-dependent variables. Ordinal least squares linear regression analyses were used to examine the association between screen time and psychological states the dependent variable. Models were adjusted for age and sex. Distributions and frequencies for each category of variables were examined, and unstandardized regression coefficients (b), standard error (SE), odds ratios (OR), and *p*-values were calculated, where appropriate, to determine the relationships between screen time and the dependent variables.

## 3. Results

### 3.1. Descriptive Characteristics 

Approximately 53% of the sample was male, nearly 58% of students were enrolled in high school (grades 10–12), and the average age was 15.1 years (SD = 1.70). Based on BMI z-score, most participants (85.26%) were considered normal weight or underweight, whilst 14.74% were classified as overweight or obese. Approximately 40% of students exceeded screen time recommendations of less than 14 h per week (<2 h per day, on average). Nearly 48% of students reported being in the top 40% in grade rankings following their final examination. Approximately 77% of students reported consuming breakfast every day, and nearly 45% of students reported snacking at night. Most students (56%) reported sleeping less than eight hours per night on school days. Only 38.7% of the participants reported strenuous activity on more than three days a week. Very few participants (4.23%) reported high levels of anxiety; however, 60.72% were dissatisfied with their lives, and 72.11% reported low self-esteem (Table 1).

#### 3.1.1. Academic Performance

After adjusting for grade and sex, more than four hours spent on social networking sites on both school days (OR = 0.412, *p* = 0.002) and non-school days (OR = 0.577, *p* = 0.001) was negatively associated with academic performance. Watching television for two to four hours (OR = 0.534, *p* = 0.027) and watching videos less than one hour on school days or not every day (OR = 0.760, *p* = 0.021) were also negatively associated with academic performance (Table 2).

#### 3.1.2. Unhealthy Eating Behaviors

Playing electronic games for less than one hour or more than 4 h on both school days and non-school days was negatively associated with frequent breakfast consumption (Table 3). On school days, watching television for two to four hours daily was positively associated with snacking at night (OR = 2.084, *p* = 0.007). On non-school days, watching television not every day, less than 1 h per day (OR = 1.346, *p* = 0.015), or more than 4 h per day (OR = 1.450, *p* = 0.017) was positively associated with snacking at night, as was playing electronic games (OR = 1.698, *p* = 0.000).

#### 3.1.3. BMI z-Score

Using social networking sites for two to four hours on non-school days was positively associated with having normal weight or being underweight (OR = 1.580, *p* = 0.028). BMI z-score categories were not associated with any other screen-based behaviors (Table 4).

#### 3.1.4. Sleep Duration

Playing e-games for two to four hours (OR = 1.307, *p* = 0.039) on non-school days and watching television not every day or less than one hour on school days (OR = 1.462, *p* = 0.001) were both positively associated with greater sleep duration. Watching videos (OR = 0.763, *p* = 0.041) not every day or less than one hour on school days was negatively associated with greater sleep duration (Table 4).

#### 3.1.5. PA

On school days, watching television for more than four hours (OR = 0.258, *p* = 0.038) was negatively associated with PA. On non-school days, using social networking sites for two to four hours daily (OR = 1.489, *p* = 0.014) was positively associated with PA (Table 4).

#### 3.1.6. Anxiety, Life Satisfaction, and Self-Esteem 

Adjusted by sex and grade, watching television on school days for two to four hours daily (b = −0.094, *p* = 0.047) was negatively associated with anxiety. Receiving electronic news or study materials for two to four hours (b = 0.062, *p* = 0.036) or more than four hours (b = 0.190, *p* = 0.002) on school days was positively associated with anxiety. Using social networking sites not every day or less than one hour on non-school days (b = 0.072, *p* = 0.009), or more than two hours on both school days and non-school days, was positively associated with anxiety. On school days, watching television for more than four hours (b = −3.825, *p* = 0.012) was negatively associated with life satisfaction. Watching television on school days for two to four hours (b = −0.935, *p* = 0.032) was negatively associated with self-esteem (Table 5).

## 4. Discussion

In this study, we found that for Chinese adolescents, more time spent watching television, on social networking sites, and videos may be negatively associated with academic performance; however, the association between academic performance and receiving news and study materials from electronic devices was not statistically significant. One possible explanation is that Chinese students may use electronic devices in “cram schools”, which may neutralize the negative effect of screen-based behaviors. Additionally, our results suggest no negative relationships between screen-based behaviors and BMI z-score. Viewing only social networking sites was significantly associated with BMI z-score, but only on non-school days. More time on such sites was associated with being underweight or normal weight. Although studies using both self-reported and objective measures of height and weight indicate that sedentary behaviors are associated with excess weight in children and adolescents [34,35], we did not observe an association between screen-based behaviors and weight status after adjustment for covariates (sex and grade).

A possible explanation for these findings is related to the associations between social networking sites and body image concerns among adolescents [36,37]. For example, Tiggemann and Slater [37] indicated that adolescent female Facebook users report more appearance concerns and dieting behavior than non-users, and that this link intensified with the amount of time spent on Facebook. Appearance comparison may explain the association between use of social network sites and lower BMI z-score. Social networking sites allow users to post photos and compare their appearance with others, placing users at a higher risk of body dissatisfaction [2]. Moreover, using SNS is not always a sedentary behavior. Students could also spend time on SNS when they are walking, for example; this may be another reason why we did not observe an association between screen-based behaviors and weight status.

Our results suggest that the amount of time reported playing electronic games and watching television is positively associated with unhealthy eating habits, although this relationship was not linear. These findings corroborate previous research indicating that sedentary behavior, particularly screen time, predicts unhealthy eating behavior [38,39]. Previous studies have suggested that the type rather than volume of sedentary behavior may be more important in explaining unhealthy eating behaviors [40,41,42]. For example, Borghese et al. [40] suggested that time spent watching TV is more strongly associated with unhealthy eating choices than total sedentary time is. Our results confirm this by showing that television viewing on school days and playing electronic games on non-school days were both related to snacking at night. Our data also suggest a link between electronic games and skipping breakfast among youth, both on school days and non-school days.

Our results suggest that higher reported time spent watching TV may be associated with greater sleep duration on non-school days. Few studies have previously shown that screen time affects sleep duration, suggesting a potentially novel finding in Chinese youth. However, playing electronic games may lead to less sleep. We found that watching television on school days and getting electronic news or study materials on non-school days were negatively associated with PA, perhaps because of displacement of other activities. However, using social networking sites was positively associated with PA. Although this is contrary to previously reported findings in western youth [9], it is consistent with the relationship we found between the use of social networking sites and BMI z-score. Our results suggest that using social networking sites may be a type of sedentary behavior not associated with low PA and a higher BMI among adolescents. Future studies are needed to better elucidate the associations between BMI, PA, and the use of social networking sites.

In this survey, anxiety levels, life satisfaction, and self-esteem levels were independently related to screen-based behaviors, especially on school days. Use of social networking sites and behaviors related to news or study materials from electronic devices were significantly associated with higher levels of anxiety in a whole week. Our results contribute to a relatively small body of literature that suggests relationships between screen-based behaviors and markers of poor mental health in adolescents. If our results were observed over a longer period of time, they could suggest greater anxiety, lower life satisfaction, and lower self-esteem in youth who spend excessive amounts of time engaged in screen-based behaviors [43], which may negatively affect their academic performance.

Childhood overweight, obesity, and insufficient PA are among the most pressing public health concerns today [44,45]. Several factors contribute to the imbalance between energy intake and energy expenditure that influence weight gain [44]. PA and screen-based behavior habits begin to form in youth [45]. Therefore, encouraging and building healthy habits regarding PA and screen-based behaviors is important to both the current and future health of children and adolescents [46,47,48].

There are some limitations to the present study. The sample is limited to one place and may not be representative of other groups of adolescents. All data were self-reported; therefore, reporting bias could have influenced the results. Assessment of PA was limited to a single item to reduce participant burden. Unfortunately, this approach allowed only for the assessment of vigorous (strenuous) PA at the exclusion of light or moderate activities, which is a limitation of the present work. That the weight status was dichotomized may have caused potential problems given that being underweight was a risk factor for certain diseases. Moreover, the cross-sectional design limits the ability to determine causal relationships. Thus, future longitudinal studies are needed. Finally, non-validated measures in our questionnaire aiming to measure the outcome variables (i.e., breakfast skipping, snacking at night, sleep duration and academic performance) may raise potential issues related to reliability. More refined and precise measures of unhealthy eating behavior would be desirable in future studies.

The strengths of this study include the large sample of Chinese adolescents, the robust data quality assurance procedures, and controlling for variables with known relationships to the dependent variables (i.e., age and gender). Unique to this analysis are scales developed for Chinese youth, previously piloted in another sample.

## 5. Conclusions

We observed differential associations between time spent on screen-based behaviors and unhealthy eating behaviors, BMI z-score, academic performance, and mental health in this sample of Chinese adolescents. If these results are indeed confirmed, Chinese school officials and policymakers should establish strategies to minimize these negative effects. However, future research is needed to better understand the impact of screen-based behaviors on health outcomes in youth.

## Figures and Tables

**Table 1:** Demographic characteristics of participating youth (*n* = 2625) and Chi-square analyses by gender for all dependent variables.

Characteristics	Whole Sample (*n* = 2625)		Male (*n* = 1394)		Female (*n* = 1231)			
	*n*	%	*n*	%	*n*	%	*Χ^2^*	*p* ^1^
Gender								
Male	1394	53.10	-	-	-	-		
Female	1231	46.90	-	-	-	-		
Grade							3.2779	0.070
Junior High School	1115	42.48	615	55.16	500	44.84		
Senior High School	1510	57.52	779	51.59	731	48.41		
Body mass index (BMI) z-score							88.9348	**0.000**
Underweight/Normal	2238	85.26	1103	49.29	1135	50.71		
Overweight/Obese	387	14.74	291	75.19	96	24.81		
Screen time (hour/week)							10.2942	**0.006**
0–7.0 h	539	22.59	309	52.11	284	47.89		
7.5–14.0 h	1102	41.98	553	50.18	549	49.82		
>14.0 h	930	35.43	532	57.20	398	42.80		
Watching TV							39.9247	**<0.001**
Never	1677	63.89	813	58.32	864	70.19		
Not every day or <1 h	855	32.57	524	37.59	331	26.89		
2–4 h	74	2.82	45	3.23	29	2.36		
>4 h	19	0.72	45	0.86	7	0.57		
Playing e-games							45.0151	**<0.001**
Never	1807	68.84	884	63.41	923	74.98		
Not every day or <1 h	687	26.17	422	30.27	265	21.53		
2–4 h	104	3.96	66	4.73	38	3.09		
>4 h	27	1.03	22	1.58	5	0.41		
Electronic news/study materials							9.1870	**0.027**
Never	1110	42.29	624	44.76	486	39.48		
Not every day or <1 h	1153	43.92	577	41.39	576	46.79		
2–4 h	303	11.54	159	11.41	144	11.70		
>4 h	59	2.25	34	2.44	25	2.03		
Using Social networking sites (SNS)							2.4643	0.482
Never	1237	47.12	668	47.92	569	46.22		
Not every day or <1 h	1056	40.23	547	39.24	509	41.35		
2–4 h	224	8.53	116	8.32	108	8.77		
>4 h	108	4.11	63	4.52	45	3.66		
Watching videos							1200	**<0.001**
Never	1617	61.60	827	59.33	790	64.18		
Not every day or <1 h	785	29.90	425	40.49	360	29.24		
2–4 h	170	6.48	103	7.39	67	5.44		
>4 h	53	2.02	39	2.80	14	1.14		
Academic performance							0.0014	0.970
First 40%	1257	47.89	668	53.14	589	46.86		
Last 60%	1368	52.11	726	53.07	642	46.93		
Frequent breakfast							0.4324	0.511
No	616	23.47	320	51.95	296	48.05		
Yes	2009	76.53	1074	53.46	935	46.54		
Snacking at night							49.3791	**0.000**
Frequently	1193	45.45	723	60.60	470	39.40		
Rarely	1432	54.55	671	46.86	761	53.14		
Typical nightly sleep							35.0139	**0.000**
<8 h	1482	56.46	712	48.04	770	51.96		
≥8 h	1143	43.54	682	59.67	461	40.33		
Physical activity (PA) ^2^							58.0788	**0.000**
Inactive	1608	61.26	759	47.20	849	52.80		
Active	1017	38.74	635	62.44	382	37.56		
Anxiety score ^3^							3.8691	0.144
6–17	2200	83.81	1174	53.36	1026	46.64		
18–23	314	11.96	171	54.46	143	45.54		
24–30	111	4.23	49	44.14	62	55.86		
Life satisfaction score ^4^							9.7196	**0.008**
5–19	1594	60.72	833	52.26	761	47.74		
20	292	11.12	180	61.64	112	38.36		
21–35	739	28.15	381	51.56	358	48.44		
Self-esteem score ^5^							6.4574	**0.040**
<15	1893	72.11	1034	54.62	859	45.38		
15–25	725	27.62	356	49.10	369	50.90		
26–30	7	0.27	4	57.14	3	42.86		

^1^ Boldface indicates statistical significance (*p* < 0.05). ^2^ Self-reported “strenuous activity” on ≥3 days in a typical week (Yes = Active; No = Inactive). ^3^ Assessed via the Middle School Student Mental Health Scale (6–17 = Low, 18–23 = Moderate, and 24–30 = Severe anxiety). ^4^ Assessed via the Satisfaction with Life Scale (5–19 = Dissatisfied, 20 = Neither Satisfied or Dissatisfied, and 21–35 = Satisfied with life). ^5^ Assessed via the Rosenberg Self-Esteem Scale (26–30 = High, 15–25 = Normal, and <15 = Low self-esteem).

**Table 2:** The relation of academic performance and screen time components (*n* = 2625).

Characteristics	Academic Performance ^1^					
	School Day Behaviors			Non-School Day Behaviors		
	OR	SE	*p* ^2^	OR	SE	*p*
Age	1.014	0.025	0.575	1.030	0.025	0.224
Gender	0.956	0.078	0.580	1.006	0.089	0.942
Watching TV						
Never	Ref.					
not every day or <1 h	0.889	0.092	0.256	1.041	0.124	0.731
2–4 h	0.534	0.152	**0.027**	0.930	0.103	0.514
>4 h	1.754	1.062	0.353	0.755	0.115	0.066
Playing e-games ^3^						
Never	Ref.					
not every day or <1 h	1.035	0.123	0.775	0.810	0.095	0.073
2–4 h	1.173	0.286	0.512	0.933	0.110	0.558
>4 h	0.402	0.250	0.143	1.005	0.149	0.971
Electronic news/study materials						
Never	Ref.					
not every day or <1 h	1.137	0.124	0.242	1.210	0.183	0.206
2–4 h	0.859	0.143	0.363	1.093	0.164	0.554
>4 h	0.955	0.354	0.902	1.043	0.181	0.809
Using SNS ^4^						
Never	Ref.					
Not every day or <1 h	1.026	0.119	0.823	0.876	0.133	0.384
2–4 h	0.700	0.131	0.056	0.798	0.122	0.142
>4 h	0.412	0.119	**0.002**	0.577	0.099	**0.001**
Watching videos						
Never	Ref.					
Not every day or <1 h	0.760	0.091	**0.021**	0.833	0.123	0.215
2–4 h	0.733	0.156	0.144	0.934	0.133	0.634
>4 h	0.956	0.379	0.911	0.864	0.146	0.388
Log Likelihood	−1782.46			−1796.7641		
Model L-squared	69.41			40.80		

^1^ 0 = 60th–100th percentile; 1 = <40th percentile. ^2^ Values in bold are statistically significance (*p* < 0.05). ^3^ e-games: Electronic games. ^4^ SNS: Social networking sites.

**Table 3:** The relation of frequent breakfast, non-snacking at night and screen time components (*n* = 2625).

Characteristics	Frequent Breakfast ^1^						Snacking at Night ^2^					
	School Day Behaviors			Non-School Day Behaviors			School Day Behaviors			Non-School Day Behaviors		
	OR	SE	*p* ^3^	OR	SE	*p*	OR	SE	*p*	OR	SE	*p*
Age	1.159	0.033	**0.000**	1.189	0.033	**0.000**	1.262	0.032	**0.000**	1.245	0.031	**0.000**
Gender	0.897	0.087	0.262	0.896	0.093	0.292	0.591	0.049	**0.000**	0.636	0.057	**0.000**
Watching TV												
Never	Ref.						Ref.					
Not every day or <1 h	0.949	0.114	0.662	1.097	0.157	0.520	1.112	0.118	0.315	1.346	0.164	**0.015**
2–4 h	1.229	0.366	0.488	1.023	0.136	0.862	2.084	0.565	**0.007**	1.059	0.121	0.617
>4 h	2.370	1.600	0.201	0.949	0.167	0.764	1.386	0.787	0.565	1.450	0.225	**0.017**
Playing e-games ^4^												
Never	Ref.						Ref.					
Not every day or <1 h	0.715	0.095	**0.012**	0.674	0.094	**0.005**	1.194	0.145	0.145	1.2313	0.148	0.084
2–4 h	1.107	0.294	0.700	0.897	0.129	0.448	1.134	0.275	0.604	1.228	0.150	0.092
>4 h	0.463	0.238	0.134	0.691	0.120	**0.033**	0.908	0.454	0.846	1.698	0.256	**0.000**
Electronic news/study materials												
Never	Ref.						Ref.					
Not every day or <1 h	1.172	0.156	0.233	1.182	0.213	0.355	0.962	0.108	0.729	0.944	0.146	0.708
2–4 h	0.824	0.150	0.291	0.978	0.174	0.901	0.770	0.130	0.122	0.872	0.134	0.373
>4 h	0.729	0.276	0.403	0.887	0.179	0.553	0.672	0.243	0.272	0.778	0.138	0.158
Using SNS ^5^												
Never	Ref.						Ref.					
Not every day or <1 h	0.900	0.126	0.454	0.882	0.163	0.495	1.028	0.123	0.820	0.898	0.146	0.708
2–4 h	0.716	0.146	0.100	0.937	0.175	0.728	1.318	0.247	0.140	0.937	0.134	0.373
>4 h	0.597	0.168	0.067	0.973	0.200	0.892	1.191	0.320	0.517	0.964	0.138	0.158
Watching videos												
Never	Ref.						Ref.					
Not every day or <1 h	0.786	0.108	0.079	0.932	0.167	0.693	1.025	0.125	0.839	0.983	0.150	0.911
2–4 h	0.841	0.191	0.445	0.855	0.147	0.362	1.431	0.300	0.087	1.148	0.169	0.349
>4 h	1.383	0.584	0.443	0.954	0.193	0.818	1.147	0.437	0.720	1.086	0.189	0.636
Log Likelihood	−1383.6966			−1399.895			−1731.0824			−1730.726		
Model L	93.09			60.69			155.07			155.78		

^1^ 0 = ate breakfast less frequently; 1 = ate breakfast on all 7 days. ^2^ 1 = frequent snacking at night; 0 = never or rarely snack at night. ^3^ Values in bold are statistically significance (*p* < 0.05). ^4^ e-games: Electronic games. ^5^ SNS: Social networking sites.

**Table 4:** The relation of BMI z-score, sleep duration, PA and screen time components (*n* = 2625).

Characteristics	BMI z-Score ^1^						Sleep Duration ^2^						PA ^3^					
	School Day Behaviors			Non-School Day Behaviors			School Day Behaviors			Non-School Day Behaviors			School Day Behaviors			Non-School Day Behaviors		
	OR	SE	*p* ^4^	OR	SE	*p*	OR	SE	*p*	OR	SE	*p*	OR	SE	*p*	OR	SE	*p*
Age	1.257	0.043	**0.000**	1.260	0.044	**0.000**	0.581	0.017	**0.000**	0.597	0.017	**0.000**	0.865	0.022	**0.000**	0.856	0.215	**0.000**
Gender	3.305	0.428	**0.000**	3.060	0.426	**0.000**	0.581	0.052	**0.000**	0.598	0.058	**0.000**	0.528	0.045	**0.000**	0.537	0.050	**0.000**
Watching TV																		
Never	Ref.						Ref.						Ref.					
Not every day or <1 h	1.034	0.148	0.815	0.928	0.159	0.663	1.462	0.167	**0.001**	1.044	0.137	0.742	1.007	0.108	0.946	0.831	0.103	0.135
2–4 h	1.234	0.437	0.552	1.022	0.165	0.892	1.443	0.415	0.202	1.130	0.139	0.320	0.930	0.248	0.785	0.994	0.115	0.956
>4 h	0.882	0.668	0.868	1.178	0.259	0.457	1.099	0.648	0.872	1.251	0.209	0.180	0.258	0.169	**0.038**	0.745	0.119	0.065
Playing e-games ^5^																		
Never	Ref.						Ref.						Ref.					
Not every day or <1 h	1.100	0.181	0.562	1.052	0.201	0.790	0.983	0.128	0.897	1.061	0.138	0.651	0.926	0.113	0.530	0.883	0.110	0.320
2–4 h	0.831	0.251	0.541	0.803	0.145	0.226	1.054	0.270	0.838	1.307	0.170	0.039	0.942	0.226	0.803	1.085	0.134	0.510
>4 h	1.826	1.267	0.386	1.010	0.217	0.964	1.110	0.580	0.842	1.128	0.183	0.459	1.901	1.041	0.240	1.000	0.154	0.997
Electronic news/study materials																		
Never	Ref.						Ref.						Ref.					
Not every day or <1 h	0.913	0.144	0.564	1.097	0.234	0.664	0.890	0.107	0.332	1.045	0.175	0.794	1.001	0.115	0.934	0.743	0.116	0.057
2–4 h	0.775	0.172	0.250	0.891	0.188	0.585	0.810	0.146	0.241	0.937	0.155	0.695	1.075	0.181	0.668	0.739	0.115	0.051
>4 h	0.593	0.279	0.266	0.899	0.215	0.655	1.034	0.387	0.928	1.000	0.189	0.998	1.423	0.509	0.324	0.805	0.143	0.223
Using SNS ^6^																		
Never	Ref.						Ref.						Ref.					
Not every day or <1 h	1.117	0.186	0.509	1.401	0.288	0.102	0.844	0.109	0.189	0.930	0.157	0.665	1.137	0.137	0.287	1.237	0.199	0.185
2–4 h	0.763	0.184	0.263	1.580	0.330	**0.028**	0.724	0.145	0.108	0.915	0.157	0.604	1.431	0.267	0.055	1.489	0.242	**0.014**
>4 h	1.663	0.649	0.192	1.454	0.336	0.106	0.791	0.223	0.406	0.760	0.144	0.147	1.282	0.341	0.350	1.223	0.222	0.264
Watching videos																		
Never	Ref.						Ref.						Ref.					
Not every day or <1 h	0.895	0.150	0.510	1.296	0.280	0.231	0.763	0.101	**0.041**	0.937	0.154	0.689	0.861	0.106	0.226	1.056	0.163	0.722
2–4 h	1.170	0.330	0.578	0.988	0.202	0.951	0.668	0.152	0.076	1.227	0.194	0.197	1.033	0.216	0.877	0.956	0.143	0.762
>4 h	0.856	0.412	0.746	0.812	0.196	0.388	0.522	0.210	0.107	0.938	0.177	0.732	1.686	0.657	0.180	0.969	0.172	0.860
Log Likelihood	−1021.5792			−1017.284			−1548.4339			−1554.1503			−1690.5321			−1690.4635		
Model L	152.51			161.10			498.25			486.82			123.75			123.89		

^1^ 0 = overweight/obese; 1 = under/normal weight. ^2^ 0 = slept <8 h a night; 1 = slept ≥8 h a night. ^3^ 0 = engaged in “strenuous activity” on <3 days in a typical week; ^4^ Values in bold are statistically significance (*p* < 0.05); ^5^ e-games: Electronic games; ^6^ SNS: Social networking sites. 1 = engaged in “strenuous activity” on ≥3 days in a typical week.

**Table 5:** The relation of anxiety, life satisfaction and self-esteem and screen time components (*n* = 2625).

Characteristics	Log Anxiety ^1^						Life Satisfaction ^2^						Self-Esteem ^3^					
	School Day Behaviors			Non-School Day Behaviors			School Day Behaviors			Non-School Day Behaviors			School Day Behaviors			Non-School Day Behaviors		
	b	SE	*p* ^4^	b	SE	*p*	b	SE	*p*	b	SE	*p*	b	SE	*p*	b	SE	*p*
Age	0.019	0.004	**0.000**	0.016	0.004	0.000	−0.404	0.067	**0.000**	−0.436	0.067	**0.000**	−0.266	0.040	**0.000**	−0.259	0.040	**0.000**
Gender	0.065	0.015	**0.000**	0.062	0.016	0.000	0.157	0.225	0.486	0.031	0.246	0.901	0.688	0.135	**0.000**	0.683	0.148	**0.000**
Watching TV																		
Never	Ref.						Ref.						Ref.					
Not every day or <1 h	−0.023	0.019	0.210	−0.033	0.022	0.131	0.238	0.287	0.407	0.360	0.331	0.277	0.169	0.173	0.327	0.259	0.199	0.192
2–4 h	−0.094	0.047	**0.047**	−0.031	0.020	0.129	0.251	0.725	0.730	0.339	0.309	0.273	−0.935	0.436	**0.032**	0.166	0.186	0.373
>4 h	−0.082	0.099	0.408	0.034	0.027	0.208	−3.825	1.520	**0.012**	−0.520	0.420	0.216	−0.383	0.913	0.675	−0.057	0.253	0.823
Playing e-games ^5^																		
Never	Ref.						Ref.											
Not every day or <1 h	−0.027	0.021	0.199	−0.033	0.021	0.118	0.310	0.329	0.346	0.177	0.325	0.587	−0.300	0.197	0.129	−0.353	0.195	0.071
2–4 h	−0.018	0.043	0.664	−0.028	0.021	0.192	−0.348	0.653	0.594	−0.096	0.328	0.770	−0.243	0.392	0.536	−0.107	0.198	0.589
>4 h	−0.167	0.088	0.058	−0.010	0.027	0.711	1.332	1.354	0.325	−0.475	0.410	0.247	0.101	0.813	0.901	−0.109	0.247	0.659
Electronic news/study materials																		
Never	Ref.						Ref.						Ref.					
Not every day or <1 h	0.012	0.020	0.542	0.052	0.027	0.059	0.529	0.304	0.082	0.411	0.420	0.328	0.110	0.183	0.548	0.446	0.253	0.078
2–4 h	0.062	0.030	**0.036**	0.041	0.027	0.135	−0.404	0.454	0.373	0.429	0.419	0.304	0.208	0.273	0.445	0.272	0.251	0.279
>4 h	0.190	0.063	**0.002**	0.060	0.031	0.055	−0.041	0.960	0.966	−0.304	0.480	0.527	−0.031	0.576	0.957	0.203	0.289	0.483
Using SNS ^6^																		
Never	Ref.						Ref.						Ref.					
Not every day or <1 h	0.037	0.021	0.078	0.072	0.028	**0.009**	−0.045	0.323	0.890	0.235	0.423	0.579	−0.126	0.194	0.516	−0.241	0.255	0.344
2–4 h	0.085	0.033	**0.010**	0.087	0.028	**0.002**	0.049	0.507	0.924	0.054	0.429	0.900	−0.178	0.304	0.559	−0.351	0.259	0.174
>4 h	0.106	0.047	**0.025**	0.087	0.031	**0.005**	−0.652	0.726	0.369	−0.037	0.477	0.938	0.052	0.436	0.905	−0.190	0.287	0.507
Watching videos																		
Never	Ref.						Ref.						Ref.					
Not every day or <1 h	0.009	0.022	0.661	0.001	0.027	0.975	−0.264	0.330	0.425	0.357	0.412	0.385	−0.062	0.198	0.754	−0.108	0.248	0.662
2–4 h	0.001	0.037	0.970	0.001	0.026	0.964	−0.232	0.570	0.684	0.405	0.397	0.308	0.067	0.342	0.845	0.044	0.239	0.853
>4 h	0.013	0.067	0.846	0.049	0.031	0.106	−0.263	1.032	0.799	0.331	0.470	0.481	0.125	0.620	0.840	0.091	0.283	0.747
F	5.08			5.57			3.89			4.29			5.03			4.92		
Adjusted R-squared	0.0257			0.0288			0.0184			0.0209			0.0254			0.0248		

^1^ Assessed via the Middle School Student Mental Health Scale with scores ranging from 6 to 30 (higher scores indicating higher levels of anxiety), then undergoing a logarithmic transformation. ^2^ Assessed via the Satisfaction with Life Scale with scores ranging from 5 to 35 (higher scores indicating higher levels of life satisfaction). ^3^ Assessed via the Rosenberg Self-Esteem Scale with scores ranging from 10 to 30 (higher scores indicating higher levels of self-esteem); ^4^ Values in bold are statistically significance (*p* < 0.05); ^5^ e-games: Electronic games. ^6^ SNS: Social networking sites.

## Appendix A

**Screen Time and Life Behaviors Questionnaire for Adolescents in Wuhan, China (School of Health Sciences, Wuhan University)**

**Basic Information**

**1. What is your sex?**

A. Male B. Female

**2. What is your date of birth? ____ (year/mm/dd)**

**3. What is your grade?**

A. Grade Seven B. Grade Eight C. Grade Nine D. Grade Ten E. Grade Eleven F. Grade Twelve

**4. Height ____ (in centimeter)**

**5. Weight ____ (in kilogram)**

**6. What is the rank of your last academic performance (including all the subjects in school) in your grade?**

A. Top 20% B. 20–40% C. 40–60% D. 60–80% E. Last 20%

**7. How many days do you usually spend on high intensive physical activities (including running, playing football, playing basketball and so on) in a week?**

A. 1–2 days B. 3 days C. 4–5 days D. Almost every day

**8. How long do you cumulatively spend on the day you do high intensive physical activities?**

A. 10–20 min B. 30–40 min C. 50–60 min D. More than an hour

**9. How long do you usually spend on sleeping on school-day?**

A. Less than 4 h B. 6 h C. 7 h D. 8 h E. 9 h F. More than 10 h

**10. How often do you have breakfast?**

A. Every day B. 4~5 days/week C. 2~3 times/week D. 1 time/week E. Never

**11. How often do you eat food late at night?**

A. Every day B. 4~5 days/week C. 2~3 times/week D. 1 time/week E. Never

**Part 1. Screen Time**

**Table A1:** How many hours do you usually spend every day on the following activities at school days and non-school days respectively?

Screen-Based Behaviors		≤1 h	2–3 h	3–4 h	>4 h	Not Everyday	Never
1 Watching television	School days						
	Non-school days						
2. Playing electronic games	School days						
	Non-school days						
3. Getting news or study materials from electronic devices	School days						
	Non-school days						
4. Using SNS (Facebook, twitter, QQ, WeChat, micro-blog, Renren)	School days						
	Non-school days						
5. Watching videos on phones or computers	School days						
	Non-school days						

**Part 2. Mental Health**

**Table A2:** Mental health scale.

**Anxiety**																
		**Never**			**Mild**			**Moderate**			**Severe**			**Strongly Severe**		
1. I feel nervous or stressed																
2. I am on tenterhooks or disconcerted																
3. I feel afraid suddenly for no reason																
4. I feel antsy																
5. I feel uncomfortable in my heart																
6. I feel uncomfortable in my heart																
7. I always have something in my mind																
**Self-Esteem**																
				**Strongly Agree**			**Agree**			**Disagree**			**Strongly Disagree**			
1. I feel that I am a person of worth, at least on an equal plane with others																
2. I feel that I have a number of good qualities																
3. All in all, I am inclined to feel that I am a failure																
4. I am able to do things as well as most people																
5. I feel that I do not have much to be proud of																
6. I take a positive attitude toward myself																
7. On the whole, I am satisfied with myself																
8. I wish I could have more respect for myself																
9. I certainly feel useless at times																
10. At times, I feel that I am no good at all																
**Happiness**																
	**Strongly Disagree**		**Disagree**			**Slightly Disagree**			**Neither Agree nor Disagree**			**Slightly Agree**			**Agree**	**Strongly Agree**
1. In most ways, my life is close to my ideal																
2. The conditions of my life are excellent																
3. I am satisfied with my life																
4. So far, I have gotten the important things I want in my life																
5. If I could live my life over, I would change almost nothing																

## References

[1] Springer E.A., Selwyn B.J., Kelder S.H. (2006). A descriptive study of youth risk behavior in urban and rural secondary school students in El Salvador. BMC Int. Health Hum. Rights.

[2] Sampasa-Kanyinga H., Chaput J.P., Hamilton H.A. (2015). Associations between the use of social networking sites and unhealthy eating behaviours and excess body weight in adolescents. Br. J. Nutr..

[3] Robinson S., Daly R.M., Ridgers N.D., Salmon J. (2015). Screen-based behaviors of children and cardiovascular risk factors. J. Pediatr..

[4] Tremblay M.S., Leblanc A.G., Janssen I., Kho M.E., Hicks A., Murumets K., Colley R.C., Duggan M. (2011). Canadian sedentary behaviour guidelines for children and youth. Appl. Physiol. Nutr. Metab..

[5] The Department of Health (2013). Australia’s Physical Activity and Sedentary Behaviour Guidelines for Young People (13–17 Years).

[6] American Academy of Pediatrics (2001). Media violence. Committee on public education. Pediatrics.

[7] Gray C.E., Larouche R., Barnes J.D., Colley R.C., Bonne J.C., Arthur M., Cameron C., Chaput J.P., Faulkner G., Janssen I. (2014). Are we driving our kids to unhealthy habits? Results of the active healthy kids Canada 2013 report card on physical activity for children and youth. Int. J. Environ. Res. Public Health.

[8] Sisson S.B., Church T.S., Martin C.K., Tudor-Locke C., Smith S.R., Bouchard C., Earnest C.P., Rankinen T., Newton R.L., Katzmarzyk P.T. (2009). Profiles of sedentary behavior in children and adolescents: The U.S. National Health and Nutrition Examination Survey, 2001–2006. Int. J. Pediatr. Obes..

[9] Syvaoja H.J., Kantomaa M.T., Ahonen T., Hakonen H., Kankaanpaa A., Tammelin T.H. (2013). Physical activity, sedentary behavior, and academic performance in Finnish children. Med. Sci. Sports Exerc..

[10] Aguilar M.M., Vergara F.A., Velasquez E.J., Marina R., Garcia-Hermoso A. (2015). Screen time impairs the relationship between physical fitness and academic attainment in children. J. Pediatr..

[11] Sharif I., Sargent J.D. (2006). Association between television, movie, and video game exposure and school performance. Pediatrics.

[12] Falbe J., Rosner B., Willett W.C., Sonneville K.R., Hu F.B., Field A.E. (2013). Adiposity and different types of screen time. Pediatrics.

[13] Raistenskis J., Sidlauskiene A., Cerkauskiene R., Burokiene S., Strukcinskiene B., Buckus R. (2015). Physical activity and sedentary screen time in obese and overweight children living in different environments. Cent. Eur. J. Public Health.

[14] Corder K., Atkin A.J., Bamber D.J., Brage S., Dunn V.J., Ekelund U., Owens M., van Sluijs E.M., Goodyer I.M. (2015). Revising on the run or studying on the sofa: Prospective associations between physical activity, sedentary behaviour, and exam results in British adolescents. Int. J. Behav. Nutr. Phys. Act..

[15] Morita N., Nakajima T., Okita K., Ishihara T., Sagawa M., Yamatsu K. (2016). Relationships among fitness, obesity, screen time and academic achievement in Japanese adolescents. Physiol. Behav..

[16] Min L., Peng D., Dan W., Ping S. (2013). Study on obesity and overweight and influencing factors among middle school students in Shijingshan District, Beijing City. Chin. J. Health Educ..

[17] Xiao H. (2005). A Study on Middle School Students’ Participation in Community Sports Activities in Beijing.

[18] Wang H. (2015). Pysical Exercise Study of Experimental Middle School Students Mental Pressure Relief to the Affiliated High School of Peking University as an Example.

[19] Sun Y. (2013). Association between Obesity, Sport and Eating Habit among the Middle School Students in Wuhan.

[20] Chen Z. (2008). Comparative Study on Mental Health between Obesity and Normal Weight Students in Junior and Senior High School.

[21] Zhao J. (2015). Trends in Overweight and Obesity among Chinese Children and Adolescents Aged 2–18 Years, 1991–2011.

[22] Cole T.J., Bellizzi M.C., Flegal K.M., Dietz W.H. (2000). Establishing a standard definition for child overweight and obesity worldwide: International survey. BMJ.

[23] Lopez-Legarrea P., Olivares P.R., Almonacid-Fierro A., Gomez-Campos R., Cossio-Bolanos M., Garcia-Rubio J. (2015). Association between dietary habits and the presence of overweight/obesity in a sample of 21,385 chilean adolescents. Nutr. Hosp..

[24] Sampasa-Kanyinga H., Willmore J. (2015). Relationships between bullying victimization psychological distress and breakfast skipping among boys and girls. Appetite.

[25] Hsu Y.W., Liou T.H., Liou Y.M., Chen H.J., Chien L.Y. (2016). Measurements and profiles of body weight misperceptions among Taiwanese teenagers: A national survey. Asia Pac. J. Clin. Nutr..

[26] Gralla M.H., McDonald S., Breneman C., Beets M.W., Moore J.B. (2016). Associations of objectively measured vigorous physical activity with body composition, cardiorespiratory fitness, and cardiometabolic health in youth: A review. Am. J. Lifestyle Med..

[27] Moore J.B., Beets M.W., Brazendale K., Blair S.N., Pate R.R., Andersen L.B., Anderssen S.A., Grontved A., Hallal P.C., Kordas K. (2017). Associations of vigorous-intensity physical activity with biomarkers in youth. Med. Sci. Sports Exerc..

[28] Helmerhorst H.J., Brage S., Warren J., Besson H., Ekelund U. (2012). A systematic review of reliability and objective criterion-related validity of physical activity questionnaires. Int. J. Behav. Nutr. Phys. Act..

[29] Wang Y.L. (2006). A summary of the researches about the factors of family influence on the children necessary to be brought up by other people. Prog. Mod. Biomed..

[30] Diener E., Emmons R.A., Larsen R.J., Griffin S. (1985). The satisfaction with life scale. J. Pers. Assess..

[31] Pavot W., Diener E. (2008). The satisfaction with life scale and the emerging construct of life satisfaction. J. Posit. Psychol..

[32] Rosenberg M. (1965). Society and the Adolescent Self-Image.

[33] Jang C.H., Joo M.C., Noh S.E., Lee S.Y., Lee D.B., Lee S.H., Kim H.K., Park H.I. (2016). Effects of hippotherapy on psychosocial aspects in children with cerebral palsy and their caregivers: A pilot study. Ann. Rehabil. Med..

[34] Tremblay M.S., Willms J.D. (2003). Is the Canadian childhood obesity epidemic related to physical inactivity?. Int. J. Obes. Relat. Metab. Disord..

[35] Kautiainen S., Koivusilta L., Lintonen T., Virtanen S.M., Rimpela A. (2005). Use of information and communication technology and prevalence of overweight and obesity among adolescents. Int. J. Obes..

[36] Meier E.P., Gray J. (2014). Facebook photo activity associated with body image disturbance in adolescent girls. Cyberpsychol. Behav. Soc. Netw..

[37] Tiggemann M., Slater A. (2013). NetGirls: The Internet, Facebook, and body image concern in adolescent girls. Int. J. Eat. Disord..

[38] Pearson N., Biddle S.J. (2011). Sedentary behavior and dietary intake in children, adolescents, and adults. A systematic review. Am. J. Prev. Med..

[39] Rehm C.D., Matte T.D., Van Wye G., Young C., Frieden T.R. (2008). Demographic and behavioral factors associated with daily sugar-sweetened soda consumption in New York City adults. J. Urban Health.

[40] Borghese M.M., Tremblay M.S., Leduc G., Boyer C., Belanger P., LeBlanc A.G., Francis C., Chaput J.P. (2014). Independent and combined associations of total sedentary time and television viewing time with food intake patterns of 9- to 11-year-old Canadian children. Appl. Physiol. Nutr. Metab..

[41] Santaliestra-Pasias A.M., Mouratidou T., Verbestel V., Huybrechts I., Gottrand F., Le Donne C., Cuenca-Garcia M., Diaz L.E., Kafatos A., Manios Y. (2012). Food consumption and screen-based sedentary behaviors in European adolescents: The HELENA study. Arch. Pediatr. Adolesc. Med..

[42] Lissner L., Lanfer A., Gwozdz W., Olafsdottir S., Eiben G., Moreno L.A., Santaliestra-Pasias A.M., Kovacs E., Barba G., Loit H.M. (2012). Television habits in relation to overweight, diet and taste preferences in European children: The IDEFICS study. Eur. J. Epidemiol..

[43] Wu X., Tao S., Zhang Y., Zhang S., Tao F. (2015). Low physical activity and high screen time can increase the risks of mental health problems and poor sleep quality among Chinese college students. PLoS ONE.

[44] Daniels S.R., Arnett D.K., Eckel R.H., Gidding S.S., Hayman L.L., Kumanyika S., Robinson T.N., Scott B.J., St Jeor S., Williams C.L. (2005). Overweight in children and adolescents: Pathophysiology, consequences, prevention, and treatment. Circulation.

[45] Hills A.P., King N.A., Armstrong T.P. (2007). The contribution of physical activity and sedentary behaviours to the growth and development of children and adolescents: Implications for overweight and obesity. Sports Med..

[46] Jimenez-Pavon D., Kelly J., Reilly J.J. (2010). Associations between objectively measured habitual physical activity and adiposity in children and adolescents: Systematic review. Int. J. Pediatr. Obes..

[47] Strong W.B., Malina R.M., Blimkie C.J., Daniels S.R., Dishman R.K., Gutin B., Hergenroeder A.C., Must A., Nixon P.A., Pivarnik J.M. (2005). Evidence based physical activity for school-age youth. J. Pediatr..

[48] Rey-Lopez J.P., Vicente-Rodriguez G., Biosca M., Moreno L.A. (2008). Sedentary behaviour and obesity development in children and adolescents. Nutr. Metab. Cardiovasc. Dis..

